# AGE-FRIENDLY COMMUNITIES: ENHANCING COMMUNITY CONNECTIONS

**DOI:** 10.1093/geroni/igz038.950

**Published:** 2019-11-08

**Authors:** Patricia A Oh

**Affiliations:** University of Massachusetts, Boston, Boston, Massachusetts, United States

## Abstract

Age-friendly communities promote active, healthy, socially connected aging. Opportunities for social connections are key for older residents to enjoy the best possible health and well-being. Communities that join the AARP Network of Age-Friendly States and Communities (AARP NAFSC) include an aging lens in eight areas of community life—social participation, respect and social inclusion, civic participation and employment, communication and information, housing, transportation, community support and health services, and outdoor spaces and buildings. By addressing factors in these eight areas, communities encourage residents to enjoy formal participation in activities and groups and informal contacts with friends, neighbors and other residents. The purpose of this exploratory study was to find out if communities that join the AARP NAFSC plan and implement changes to enhance social connectedness. A review of 62 AARP-approved action plans nationwide, showed that social connectedness was included in 74% of the mission statements and was a goal in 92% of the plans. The lack of resources in rural communities creates special challenges; many age-friendly initiatives depend on community volunteers to implement changes on a shoe-string budget. To learn how rural age-friendly communities promote social connections, an email survey was distributed to 46 AARP NAFSC communities in rural Maine. All the communities responded. Fostering social connectedness was an explicit goal for 88% of the communities. Areas of implementation included services and activities (83%), communication (61%), transportation (30%), programming to include isolated residents (26%), accessible public spaces (22%), and intergenerational volunteering (17%). Implications will be discussed.

